# Reoperation risk by subtype in proximal junctional kyphosis and the impact of osteoporosis treatment in adult spinal deformity surgery

**DOI:** 10.1016/j.xnsj.2025.100821

**Published:** 2025-11-09

**Authors:** Tetsuro Ohba, Nobuki Tanaka, Kotaro Oda, Hayato Takei, Goto Go, Hirotaka Haro

**Affiliations:** Department of Orthopaedic Surgery, University of Yamanashi, Yamanashi 409-3898, Japan

**Keywords:** Proximal junctional kyphosis, Adult spinal deformity, Reoperation, Teriparatide, Bone mineral density, Osteoporosis treatment, Spinopelvic alignment, Long‑segment fusion

## Abstract

**Background:**

Mechanical complications after long-segment fusion for adult spinal deformity (ASD) remain a major driver of reoperation. Proximal junctional kyphosis (PJK) comprises heterogeneous morphologic subtypes with potentially distinct clinical courses, yet how subtype relates to reoperation risk and whether bone quality or osteoporosis medications mitigate that risk are not fully defined.

**Methods:**

We conducted a single-center retrospective cohort study of consecutive older adults undergoing posterior instrumented fusion for ASD. Patients were classified by PJK subtype and grade using standardized radiographic criteria, and followed longitudinally for the occurrence and timing of reoperation. Bone quality surrogates and osteoporosis therapies, including preoperative teriparatide, were recorded. Multivariable models adjusted for demographic, surgical, and spinopelvic parameters were used to assess associations between subtype, bone quality, medication exposure, and reoperation.

**Results:**

Reoperation clustered within specific PJK subtypes, with higher-grade deformity demonstrating a disproportionately greater need for revision. Subtypes characterized by structural failure and junctional collapse showed the strongest association with reoperation compared with alignment-predominant patterns. Lower bone quality correlated with more severe PJK and reoperation, whereas exposure to osteoporosis medication—particularly preoperative teriparatide—was associated with a lower likelihood of reoperation and delayed time to reintervention. Sensitivity analyses yielded consistent effect directions across age, sagittal alignment, and construct length strata.

**Conclusions:**

PJK subtype meaningfully stratifies reoperation risk after ASD surgery. Integrating subtype with bone quality assessment can refine perioperative decision-making, identify patients who may benefit from intensified junctional protection, and inform surveillance. Preoperative anabolic therapy emerges as a potentially modifiable factor linked with reduced reoperation, warranting prospective evaluation. These findings support a practical, subtype-guided strategy that couples surgical planning with bone health optimization to reduce failure and reoperation burden.

## Introduction

Adult spinal deformity (ASD) surgery has advanced markedly over recent years; however, mechanical complications remain a major concern, with proximal junctional kyphosis (PJK) among the most prevalent and impactful. PJK occurs in up to 20%–40% of cases following long spinal fusion (generally defined as constructs spanning ≥4 vertebral levels), with ∼20% of these patients ultimately requiring revision surgery due to progressive kyphosis, implant failure, or intractable pain [[1], [2], [3]]. PJK has been linked to decreased health-related quality-of-life, sagittal imbalance, and limited walking ability [[4], [5], [6], [7]]. Although numerous risk factors have been proposed, including excessive postoperative correction, upper thoracic fusion, older age, and paraspinal muscle loss, no definitive preventive strategy has been established [[8], [9], [10]]. Surgical techniques, such as transition rods, prophylactic hooks, and ligament augmentation, have been explored, but none have shown consistent efficacy across patient populations [11,12]. These challenges underscore the complexity of PJK pathogenesis and the need to identify modifiable risk factors.

Emerging evidence suggests that bone quality is critical to postoperative outcomes in ASD surgery. Osteoporotic bone is associated with higher risks of proximal junctional failure, pedicle screw loosening, rod fracture, and pseudarthrosis. Consequently, bone-modifying agents have garnered attention as a means of improving surgical outcomes. For example, teriparatide, a recombinant parathyroid hormone analog, has been shown to promote spinal fusion and reduce complications (eg, implant loosening and PJK) in preclinical and clinical studies [13]. The roles of denosumab and romosozumab, which act via different mechanisms on bone metabolism, have also been reported [14]. Nevertheless, it remains unclear whether the type or timing of osteoporosis treatment affects PJK risk or the need for revision surgery in clinical practice.

Although often treated as a single complication, PJK is radiologically and clinically heterogeneous. Classification systems categorize PJK by failure mechanism, including ligamentous failure, vertebral fracture, and implant-related causes. A grading system based on angular progression severity has also been associated with clinical outcomes [3]. However, few studies have examined how PJK subtypes or grades relate to the likelihood of revision surgery. To improve perioperative management and personalize treatment strategies, it is essential to acknowledge that not all PJKs have equal clinical impact.

The association between spinopelvic alignment and PJK has been reported repeatedly by our group, and similar trends were observed in the present cohort; however, in this study we prioritized analyses of PJK subtype and the need for revision surgery, together with the role of osteoporosis treatment.

This study had 2 objectives. First, we aimed to determine whether specific PJK subtypes or grades are associated with a higher likelihood of reoperation following ASD surgery. Second, we evaluated whether osteoporosis medications, particularly the type and timing of administration, affect the incidence and severity of PJK or the need for revision surgery.

## Methods

### Study design and patient selection

This retrospective cohort study included patients aged >60 years old, diagnosed with ASD characterized by sagittal or coronal imbalance, who underwent corrective spinal fusion at a single center between April 2016 and March 2023. The study was approved by the Institutional Review Board (No. 1183), and informed consent was obtained from all participants. Surgical indications included structural ASD unresponsive to prolonged (≥6 months) conservative management.

Only patients with de novo degenerative deformities were included.

### Surgical procedures

Surgical correction involved a lateral interbody fusion at L1–L5 via an anterior approach, followed by posterior lumbar interbody fusion at L5–S1 after repositioning the patient into the prone position. Sagittal realignment was achieved using a cantilever technique with bilateral S1 screws and supplemental iliac fixation. Postoperative bracing was not part of a standardized protocol and, if used, was at clinician discretion; bracing practices were not uniformly recorded and were not analyzed. No protocolized proximal junctional tethering or ligament augmentation was employed.

Osteotomies were performed as needed for limited spinal flexibility, categorized by the Scoliosis Research Society-Schwab osteotomy classification (grades 1–6). Fusion material included autologous and allograft bone; bone morphogenetic proteins were not used.

### Radiographic evaluation

Full-spine lateral radiographs were obtained preoperatively, at 4–6 weeks postoperatively, and at the 2-year follow-up, with patients in a freestanding posture and their hands placed on the clavicles. Parameters measured included thoracic kyphosis (T5–T12), thoracolumbar kyphosis (T10–L2), lumbar lordosis (T12–S1), pelvic incidence, PT, sacral slope, SVA, T1 pelvic angle, and global tilt. The lordosis distribution index was derived from the L1–S1 and L4–S1 angles.

PJK was defined as a postoperative increase in the proximal junctional angle (PJA) of ≥10° and at least 10° greater than the preoperative measurement, following the criteria proposed by Yagi et al. [3]. PJK was also classified by failure mechanism into 3 types: Type 1: soft tissue and ligamentous failure; Type 2: vertebral fracture; Type 3: implant or bone–implant interface failure. Severity was graded based on angular progression: Grade A: PJA increase of 10°–19°; Grade B: PJA increase of 20°–29°; Grade C: PJA increase ≥ 30°. Patients who did not meet radiographic criteria for PJK were classified as Type 0, and Grade 0 indicates a proximal junctional angle increase < 10° (ie, no PJK).

Radiographs were independently evaluated by 2 board-certified spine surgeons (blinded to clinical data), with their average used for analysis. Inter-rater reliability was confirmed by an intraclass correlation coefficient of 0.882.

### Statistical analysis

Continuous variables were presented as means ± standard deviations (SD), whereas categorical variables were shown as counts and percentages. The Shapiro–Wilk test was used to assess data normality. For comparisons between 2 groups, the Student’s t-test was applied to normally distributed data, whereas the Mann–Whitney U test was used for nonparametric data. For comparisons among more than 2 groups, one-way analysis of variance (ANOVA) was employed, followed by Tukey’s test applied posthoc. The chi-square test or Fisher’s exact test was used to compare categorical variables as appropriate. The relationship between BMD and complication types was assessed via one-way ANOVA and Tukey’s posthoc test.

All statistical analyses were performed using GraphPad Prism version 8.0 (GraphPad Software, Boston, MA, USA). A 2-sided p-value <.05 was considered statistically significant, and the Bonferroni correction was applied for multiple comparisons when appropriate.

## Results

### Patient characteristics

**Fig. 1:**
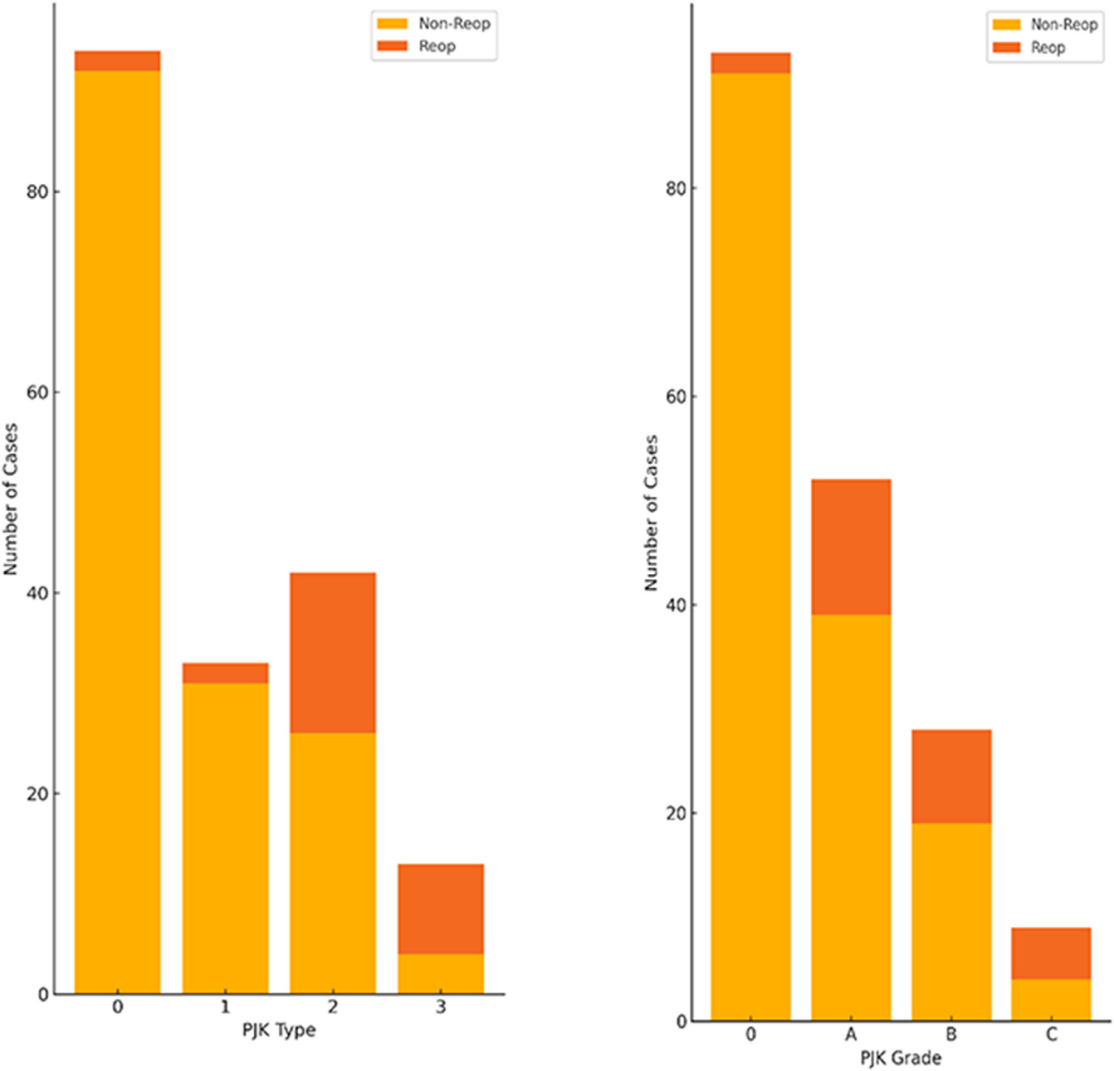
Distribution of PJK types (0–3) and grades (0–C) among patients who underwent ASD surgery (*n* = 189). Bar height reflects case frequency; red segments represent cases requiring revision surgery. Reoperation rates were significantly higher in Types 2 and 3 compared with Types 0 and 1 (p < .0001).

In total, 189 patients who underwent surgery for ASD were included. The mean age was 72.1 ± 7.4 years, and 88.9% of patients were female (*n* = 189). The average body mass index was 24.1 ± 2.8 kg/m², and the average BMD, expressed as a percentage of the young adult mean (%YAM), was 74.5% ± 15.6%. The upper instrumented vertebra was at T9–T11 in 145 cases (76.7%) and at T8 or above in 44 cases (23.3%) (Fig. 1).

### Osteoporosis medication use

Medication data were available for 182 of 189 patients (96.3%). At the time of surgery, 31.9% (*n* = 58) were not receiving bone‑modifying agents. Overall, teriparatide was used in 53.3% (*n* = 97/182), denosumab in 4.4% (*n* = 8/182), romosozumab in 4.4% (*n* = 8/182), selective estrogen receptor modulators in 3.3% (*n* = 6/182), and bisphosphonates in 2.7% (*n* = 5/182). Preoperative teriparatide administration was documented in 65 patients (mean 4.6 ± 3.5 months), and 32 patients received teriparatide postoperatively only. Preoperative duration for denosumab was 8.4 ± 3.3 months (*n* = 5). Preoperative duration for romosozumab was 4.0 ± 2.6 months (*n* = 3). Preoperative duration for bisphosphonates was 14.5 ± 13.4 months (*n* = 2) (Table 1).

**Table 1:** Baseline characteristics of patients with ASD (*N* = 189).

Variable	Mean ± SD or n (%)
Age (years)	72.1 ± 7.4 (50–85)
Female/Male (n, %)	168/21 (88.9%/11.1%)
BMI (kg/m²)	24.1 ± 2.8 (20.6–26.5)
BMD (%YAM)	74.5 ± 15.6 (46–117)
Location of UIV (n, %) — T9–T11	145 (76.7%)
Location of UIV (n, %) — T8 or above	44 (23.3%)

ASD, adult spinal deformity; BMI, body mass index; BMD, bone mineral density; YAM, young adult mean; UIV, upper instrumented vertebra.

### Changes in spinopelvic parameters: preoperative, postoperative, and 2-year follow-up

Spinopelvic parameters significantly improved postoperatively compared to the preoperative values. However, at 2 years postoperatively, a significant loss of SVA correction due to PJK was observed (Table 2).

**Table 2:** Preoperative, postoperative, and 2-year postoperative spinopelvic parameters.

Variable	Preoperative	Postoperative	2 years	p*	p†
PT (°)	38.1 ± 12.0	15.3 ± 9.1	16.1 ± 10.1	<.05	.39
SS (°)	13.0 ± 13.2	31.7 ± 8.3	29.3 ± 10.0	<.05	.09
LL (°)	6.0 ± 23.4	53.3 ± 12.1	51.1 ± 12.1	<.05	.34
PI–LL (°)	44.2 ± 20.4	−6.1 ± 12.1	−4.1 ± 10.1	<.05	.39
SVA (mm)	136.1 ± 70.1	20.4 ± 36.1	46.6 ± 38.1	<.05	<.05
GT (°)	56.1 ± 18.3	15.8 ± 10.2	22.0 ± 13.1	<.05	<.05
TPA (°)	44.1 ± 15.3	15.1 ± 11.4	17.1 ± 10.6	<.05	.14

Interval and ratio values are presented as mean ± standard deviation.

⁎ p < 0.05 preoperative vs. postoperative.

† p < 0.05 postoperative vs. 2 years postoperative.

### Incidence of PJK and reoperation rates

**Fig. 2:**
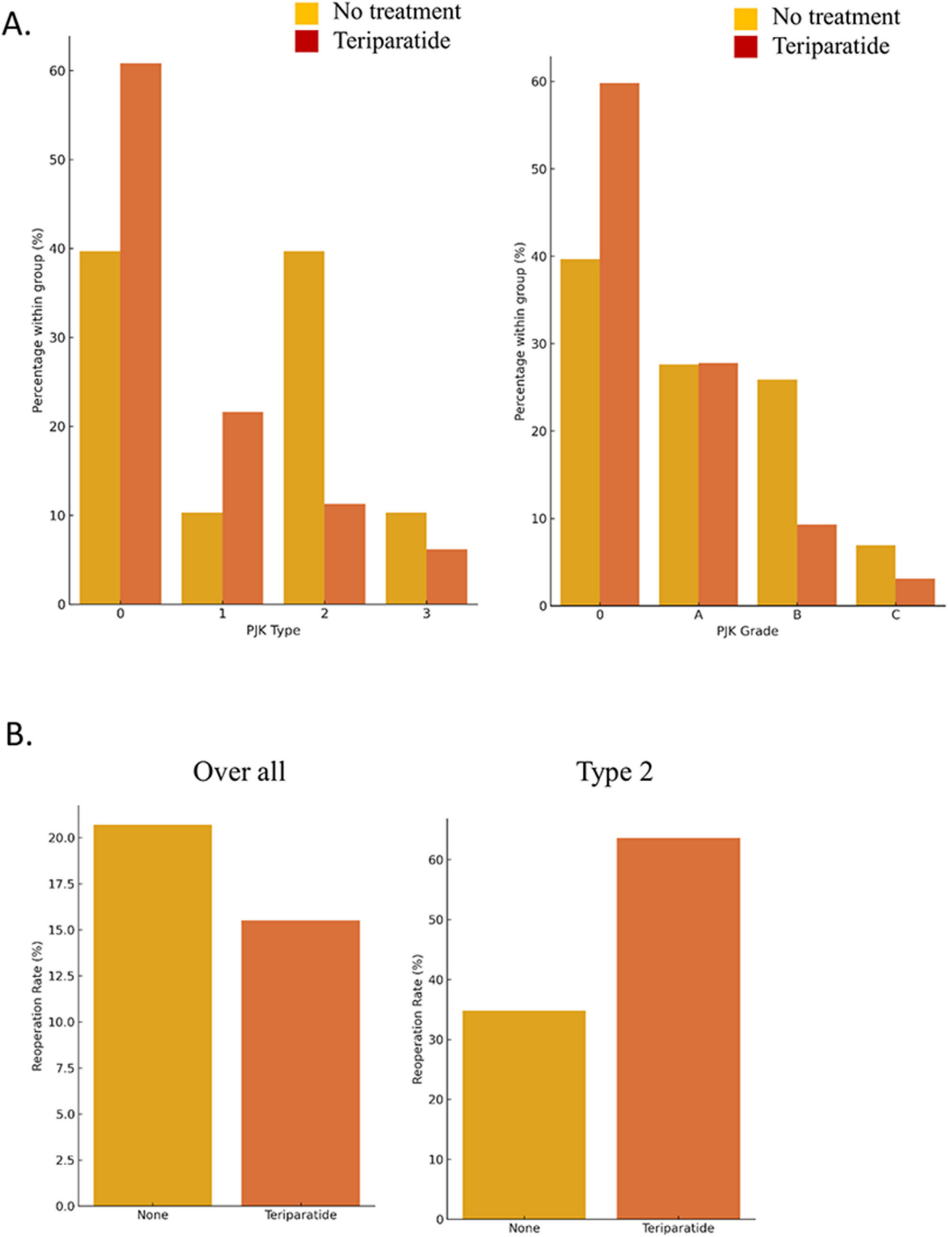
Reoperation rates according to PJK grade. Compared with Grade 0, Grades B and C showed significantly higher revision rates (p < .0001). PJK type distribution differed significantly between the teriparatide-treated and untreated groups, with a lower incidence of Type 2 PJK in the former (p = .0002). Reoperation rate by osteoporosis treatment group. The revision rate did not differ significantly between the teriparatide-treated and untreated groups (p = .5410). Among patients with Type 2 PJK, the teriparatide group had a nonsignificantly higher revision rate (Fisher’s exact test: p = .1512).

PJK occurred in 95 of 189 patients (50.3%). Distribution by PJK type was Type 0, 1, 2, and 3 in 94 (49.7%), 33 (17.5%), 42 (22.2%), and 13 (6.9%) cases, respectively. Reoperation rates varied significantly by PJK type (p < .0001). Type 3 had the highest reoperation rate (69.2%), followed by Type 2 (38.1%), Type 1 (6.1%), and Type 0 (2.1%) (Fig. 2). A similar trend was observed when stratified by PJK grade (p < .0001). Reoperation occurred in 2.2%, 25.0%, 32.1%, and 55.6% of Grade 0, A, B, and C cases, respectively (data not shown).

### BMD: association with PJK and reoperation

Although patients who underwent reoperation had a lower mean BMD compared with those who did not (70.2 ± 13.2 vs. 75.5 ± 13.5%YAM), the difference was not statistically significant (p = .1940). Similarly, a difference that did not reach statistical significance toward lower BMD was observed in patients with PJK compared to those without (73.0 ± 13.8 vs. 78.4 ± 14.0%YAM, p = .0543) (Table 3).

**Table 3:** Osteoporosis medication use and reoperation by group.

Medication	Patients, n (%)	Preoperative duration (months)	Reoperation, n (%)
Teriparatide	97 (53.3%)	4.6 ± 3.5	15 (15.5%)
None	58 (31.9%)	—	12 (20.7%)
Denosumab	8 (4.4%)	8.4 ± 3.3	—
Romosozumab	8 (4.4%)	4.0 ± 2.6	—
SERM	6 (3.3%)	12.2 ± 11.2	—
Bisphosphonate	5 (2.7%)	14.5 ± 13.4	—

Medication data were available for 182 of 189 patients (96.3%). “Preoperative duration” summarizes only patients who received the medication preoperatively; postoperative-only users are excluded from the duration calculation. Reoperation counts for Teriparatide and None were computed from cohort-level rates; other medication groups were not reported due to small sample sizes.

Stratified by PJK type, a significant difference in BMD was found (p = .0082, one-way ANOVA). Posthoc analysis revealed that patients with Type 2 PJK had significantly lower BMD compared to those with Type 0 (p = .0141), whereas no significant differences were observed between Type 2 and the other types (Table 4).

**Table 4:** Comparison of bone mineral density (BMD, %YAM) by reoperation and PJK status.

Comparison	Group 1	Group 2	p-value
Reoperation: yes vs. no	69.1 ± 12.8	75.6 ± 14.3	.0445
PJK: present vs. absent	70.3 ± 13.4	78.1 ± 14.1	.0008

PJK, proximal junctional kyphosis; BMD, bone mineral density; YAM, young adult mean.

Among 42 patients with Type 2 PJK, the reoperation rate of 38.1% was the second highest among all types. Additionally, Type 2 cases in the lowest BMD tertile had numerically higher but nonsignificant reoperation rates.

### Effect of teriparatide on PJK type and grade

A subgroup analysis comparing patients treated with teriparatide with those who received no osteoporosis medication revealed significant differences in PJK type distribution (p = .0002; Fig. 2). The teriparatide-treated group had a higher proportion of Type 0 (59 cases) and Type 1 (21 cases), with fewer Type 2 (11 cases) and Type 3 (6 cases), compared to the group receiving no osteoporosis medication (Type 2: 23 cases; Type 3: 6 cases).

Similarly, PJK grade distribution differed significantly between the groups (p = .0153; Fig. 2). The teriparatide-treated group had more Grade 0 and fewer Grades B and C cases, suggesting a potential benefit of the drug in reducing PJK severity.

### Reoperation rate based on teriparatide administration

**Fig. 3:**
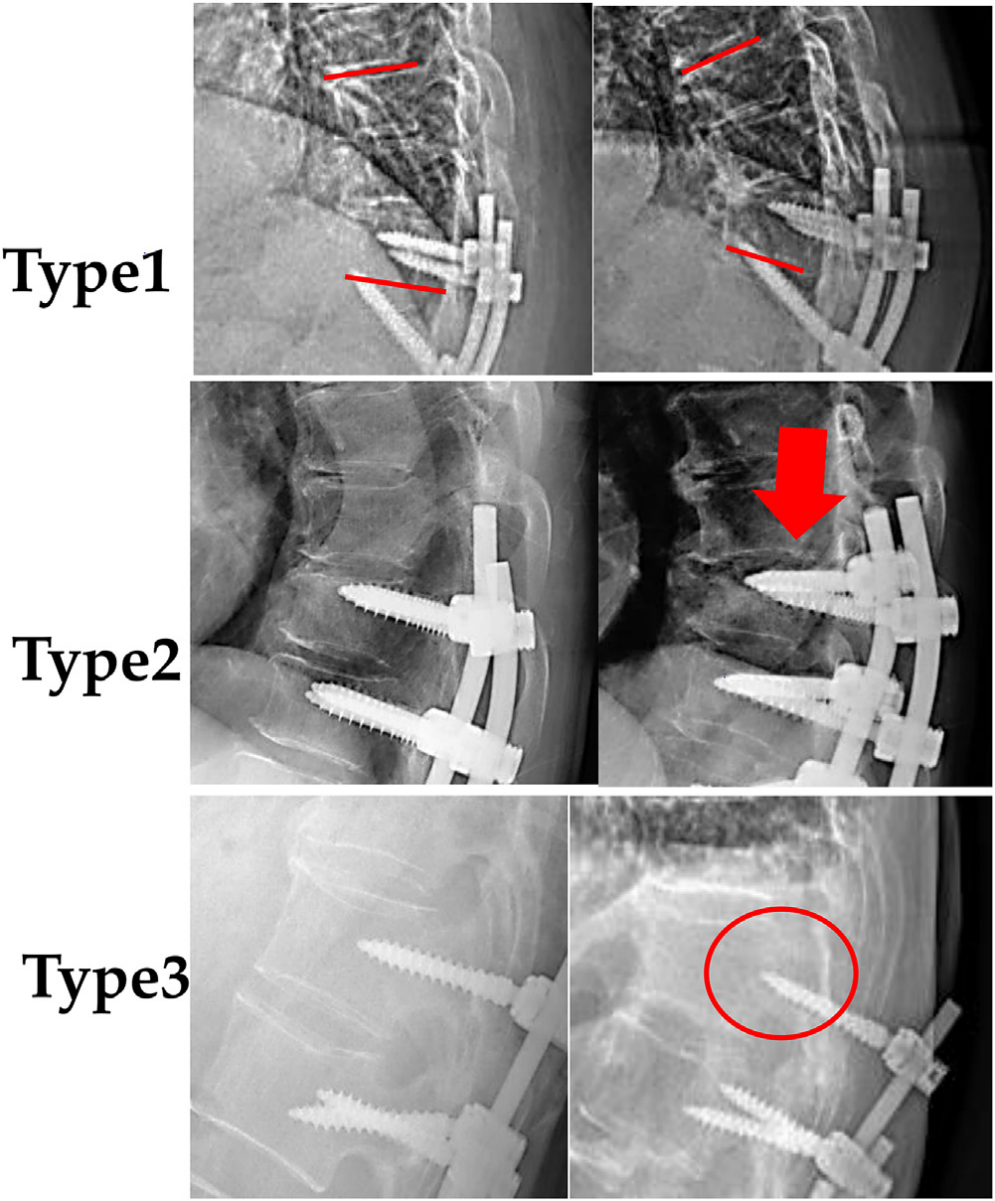
Representative lateral radiographs illustrating 3 patterns. Type 1: red lines indicate an increase in the proximal junctional angle (PJA). Type 2: red arrow denotes bony proximal junctional kyphosis (PJK). **T**ype 3: red circle highlights pedicle screw pullout. *Abbreviations:* PJA, proximal junctional angle; PJK, proximal junctional kyphosis.

The reoperation rate was 20.7% and 15.5% in the groups receiving no osteoporosis medication and teriparatide, respectively (Fig. 2), with no significant difference detected (p = .5410). Among patients with Type 2 PJK, the reoperation rate was higher in the teriparatide group (63.6%, 7 of 11 cases) than in the group receiving no osteoporosis medication (34.8%, 8 of 23 cases; Fig. 2), although the difference was nonsignificant (p = .1512, Fisher’s exact test) (Fig. 3).

### Impact of teriparatide timing on reoperation

Patients were also grouped by the timing of teriparatide administration: preoperatively, postoperatively only, and no administration. The reoperation rate was lowest in the preoperatively group (13.8%), followed by the postoperatively only (18.8%), and the no administration (20.7%) groups, although no statistically significant difference was observed.

## Discussion

In this retrospective cohort study of patients undergoing long spinal fusion for ASD, we identified several key findings regarding the relationship between PJK subtype, BMD, and osteoporosis treatment. First, we found that patients with Type 2 and Type 3 PJK were associated with significantly higher reoperation rates compared to those with Types 0 and 1, underscoring the prognostic value of subclassifying PJK beyond its presence or absence. Second, lower BMD was associated with more severe PJK subtypes, particularly Type 2, with a difference that did not reach statistical significance toward increased revision also observed. Although prior studies have suggested a link between bone quality and mechanical failure, our findings emphasize the importance of routine preoperative BMD screening and risk stratification in ASD surgery planning. Third, although teriparatide use did not significantly reduce the overall reoperation rate, patients receiving it preoperatively had numerically fewer revisions than those treated postoperatively or not at all. These findings suggest that both the use and timing of bone-modifying agents may influence surgical outcomes, warranting further prospective investigation.

Our results align with prior studies highlighting worse outcomes in structural PJK types, such as vertebral fractures (Type 2) and implant-related failures (Type 3). These forms have been linked to poorer clinical outcomes and higher revision rates compared with ligamentous failure or angular deformities without collapse. Similarly, hardware failure at the proximal junction has consistently predicted reoperation [15,16].

Although some studies have emphasized angular progression (ie, PJA) in assessing PJK, morphological classification may better reflect the mechanical instability and clinical implication of each subtype. Our findings support an integrated approach using both angular and structural criteria in postoperative surveillance and risk prediction.

The beneficial effects of anabolic agents, such as teriparatide, on spinal fusion and implant integrity have been well-documented in short-segment fusion [17,18]. Thus, there is a growing consensus that anabolic therapy enhances early postoperative spinal stability in patients with osteoporosis. However, the role of such agents in long-segment fusion for ASD remains unclear. Surgical correction for ASD often involves extensive fixation and alignment changes, with outcomes dependent on multiple interrelated factors, including sagittal balance restoration, fusion range, and baseline bone quality. Although numerous studies have addressed the elevated risk of PJK and failure in osteoporotic patients undergoing ASD surgery, standardized pharmacologic prevention strategies have yet to be established. Prospective studies have begun to fill this gap. Perioperative administration of teriparatide has been shown to reduce PJF incidence more effectively relative to antiresorptive agents, such as denosumab, although its efficacy in preventing overall PJK remains uncertain. Notably, preoperative use of teriparatide has been associated with improvements in trabecular bone microarchitecture and a reduction in vertebral fracture-type PJK [13,14,[19], [20], [21], [22]]. These data suggest that anabolic agents may play a valuable role in mitigating structural complications, particularly those related to bone fragility.

Our findings align with previous studies: patients who received teriparatide exhibited a significantly lower incidence of Type 2 PJK and a numerically but nonsignificantly reduced reoperation rate. However, no significant differences were observed among other PJK types. These results indicate that the benefit of osteoporosis therapy in ASD may depend not only on the presence of treatment but also on its timing and the biomechanical characteristics of the complication. Prospective studies stratified by complication subtype and treatment timing are warranted to validate these trends.

A key strength of this study lies in its detailed characterization of PJK subtypes and their differential impact on reoperation risk. By subclassifying PJK by morphologic type and angular grade, we showed that not all PJKs have equivalent clinical implications, particularly highlighting the structural vulnerability and clinical severity of Type 2 lesions. Moreover, this study is among the few to integrate radiographic and pharmacologic variables, including BMD and osteoporosis medication timing, into the evaluation of postoperative mechanical complications following long-segment ASD surgery.

Despite these strengths, several limitations must be acknowledged. First, the retrospective design and single center setting may limit the generalizability of our findings. Second, although we stratified patients by medication type and timing, the exact duration and adherence to preoperative therapy, especially with agents such as teriparatide, could not be consistently verified. Given emerging evidence that treatment efficacy may be dose- and time-dependent, this represents a critical area for further prospective investigation. Third, we did not assess patient-reported outcomes, such as pain or health-related quality-of-life, limiting our ability to correlate radiographic findings with functional endpoints.

Future studies should aim to validate these findings in larger, multicenter cohorts and incorporate stratified analyses based on PJK subtype, baseline bone density, and treatment duration. Establishing the optimal preoperative preparation duration of anabolic therapy may help inform standardized osteoporosis management protocols tailored to patients with ASD at high risk of mechanical failure.

## Funding

The manuscript submitted does not contain information about medical device(s)/drug(s). We did not receive any specific grant from funding agencies in the public, commercial, or not-for-profit sectors for this research.

## Ethics approval and consent to participate

The study was approved by our institutional review board (application No. 1183). The requirement for written informed consent was obtained for all participants.

## Data availability

The data are available upon request and can be discussed with the corresponding author.

## Author contributions

Conceptualization, T.O. and H.H.; Methodology, N.T, T.H and G.G. Validation, K.O, T.N and T.O.; Formal Analysis, T.O and K.M; Investigation, H.H.; Writing – Original Draft Preparation, T.O.; Writing – Review & Editing, N.T and T.O.

## Declaration of competing interest

The authors declare that they have no known competing financial interests or personal relationships that could have appeared to influence the work.
